# Re-emergence of porcine epidemic diarrhea virus in a piglet-producing farm in northwestern Germany in 2019

**DOI:** 10.1186/s12917-020-02548-4

**Published:** 2020-09-10

**Authors:** Claudia Karte, Nadine Platje, Johannes Bullermann, Martin Beer, Dirk Höper, Sandra Blome

**Affiliations:** 1 Institute of Diagnostic Virology, Friedrich-Loeffler-Institut, Federal Research Institute for Animal Health, Suedufer 10, 17493 Greifswald – Insel Riems, Greifswald, Germany; 2 Tierarztpraxis Wietmarschen, Wietmarschen, Germany; 3 Tierarztpraxis Bethen, Cloppenburg, Germany

**Keywords:** Porcine epidemic diarrhea virus (PEDV), Next-generation sequencing, Phylogenetic analysis, Metagenomics

## Abstract

**Background:**

Porcine epidemic diarrhea (PED) is a viral enteric disease of pigs. It affects all age classes of animals but lethality is mainly seen in suckling piglets. After its first appearance in England in 1971, Porcine epidemic diarrhea virus (PEDV) has spread worldwide. While sporadic outbreaks prevailed in Europe, the disease had high impact in Asia. Following particularly severe outbreaks in 2011, high impact cases were also reported in the United States and neighboring countries in 2013. Subsequently, outbreaks were also reported in several European countries including Germany. These outbreaks were less severe. This case report describes a recent case of PED re-emergence in Germany and the sequence analyses of the causative PEDV.

**Case presentation:**

In spring 2019 5 years after re-introduction of PED into Central Europe, a piglet-producer in northwestern Germany experienced an outbreak that affected sows, their suckling piglets, and weaners. After initial confirmation of PEDV by real-time RT-PCR, fecal material and small intestine samples from affected pigs were subjected to metagenomic analyses employing next-generation sequencing. Phylogenetic analyses showed high identities among the PEDV sequences obtained from samples of different animals and a close relation to recent strains from Hungary and France. Compared to the PEDV strains analyzed in 2014, genetic drift could be confirmed. Changes were mainly observed in the spike protein encoding S gene segment. In addition, metagenomic analyses showed multiple Picobirnavirus reads in all investigated samples.

**Conclusion:**

This case report shows that PEDV is still circulating in Europe. The causative strains are moderately virulent and are still closely related to the so-called INDEL strains reported previously in Europe, including Germany. However, a genetic drift has taken place that can be seen in a novel cluster comprising strains from Germany, Hungary and France in 2019. Relevance and impact of the detected Picobirna sequences need further investigations.

## Background

Porcine epidemic diarrhea (PED) is an acute and highly contagious enteric disease of swine that results in severe enteritis, diarrhea, vomiting, and dehydration. Especially in suckling pigs, lethality can be very high [1–3]. The causative agent, Porcine epidemic diarrhea virus (PEDV), is an enveloped positive single-stranded RNA virus that belongs to the family *Coronaviridae*, genus *Alphacoronavirus* [4]. The complex coronavirus particles are pleomorphic and possess club-shaped surface projectors [5]. The length of the genome ranges from 27 to 31 kilobases [6]. Coronaviruses have a low tenacity [7] but are shed in high amounts and are thus easily transmitted by the fecal-oral route [8]**.**

After its first recognition in the 1970s in Europe [7, 9], the disease caused considerable economic losses especially in Asia, where the disease remains endemic [10, 11]. In Europe, the disease disappeared quickly, and from most countries, only very sporadic cases were reported over the last three decades. After reports from Asia, that a new PEDV variant caused considerable losses [12, 13], that highly virulent PEDV variant emerged also in the United States (US) in 2013, with swine farms experiencing explosive epidemics affecting all age classes of animals, with up to 95% mortality in suckling pigs [2, 14].

In 2014, several cases of PED were also reported from southern and western Germany. In most cases, fattening pigs were affected showing high morbidity with almost non-existent mortality [15]. However, some breeding herds reported high mortality rates with up to 85% losses in suckling piglets [1]. Similar outbreaks were observed in several other Central European countries including France, The Netherlands, Italy, Slovenia, Belgium, Romania, Portugal, Spain, and Austria [16–23].

The characterization of the involved virus strains revealed that so-called S-INDEL variants of the virus were involved in central Europe, which, in contrast to the highly virulent NON-INDEL strains from Asia and the USA, are characterized by deletions and insertions in the spike protein encoding S gene [23]. In the majority of cases, the S-INDEL variants are associated with milder PED courses.

In the absence of reporting obligations, and following the confirmation that the PEDV strains in the EU did not belong to the highly virulent NON-INDEL type, notification and broader follow-up of cases decreased. However, sporadic outbreaks, sometimes with severe problems to get rid of the disease, were still reported from all production systems from different regions of Germany and other countries (personal communications and unpublished data). From this time, PEDV sequence information is largely missing.

When a new wave of PED struck a piglet producer in northwestern Germany in 2019, questions were raised to what extent the virus might have changed and if a new emerging variant was causing the clinical case in sows, piglets and weaners. Here, we report on the clinical presentation and the whole-genome sequencing of the causative 2019 PEDV strain.

## Case presentation

### Case farm characteristics

The affected piglet producer is located in northwestern Germany. The farm keeps approximately 350 sows in seven groups (50 sows each), and has a total of 2200 piglet rearing places (1000 on site and 1200 in a leased farm). On the premise, sows and piglets are kept in the same building complex. The farm also includes a fattening unit with 1500 fattening slots. This unit is in close proximity to the above-mentioned units but has its own building with a hygiene lock. Three other pig farms are in the radius of 500 m around the holding.

Prior to the disease event, the farm recorded 33 weaned piglets per sow and year with suckling losses below 10%. Loss in piglet rearing and fattening was < 2%. The animals were routinely screened for enteric pathogens and only rotavirus types A and C were detected every now and again (rotavirus type C only very sporadically). The farm was unsuspicious for dysentery and was tested negative for TGEV and PDCoV prior, during and after the disease event.

The routinely applied immunization scheme included maternal vaccinations against colibacillosis, oedema disease, and necrotic enteritis. Depending on the infection pressure, rotavirus A vaccines were used in gilts. Piglets received vaccinations against porcine circovirus type 2 (PCV-2), mycoplasma, and Shiga toxin producing *E. coli*. Sows in integration and reproduction received additional vaccination e.g. against porcine respiratory and reproductive syndrome virus, influenza virus, and parvovirus.

### Clinical presentation and interventions

In spring 2019, massive diarrhea occurred in sows and suckling pigs. At first, nursing sows (60% of the sows in the unit) in the farrowing unit showed inappetence and shortly afterwards mushy diarrhoea. Fever or increased temperature were not detected. The sows recovered completely after three to 4 days. The suckling piglets, which were about 14 days old, showed the first signs of diarrhea two to 3 days after the mothers. About 70% of the litters of this first affected farrowing group showed diarrhea and losses rose to 10% (see Table 1). Immediate investigations confirmed PEDV (RT-qPCR from fecal samples and organs). Sick piglets were treated with commercial electrolyte solution and additional water supply was given to sows. To increase maternal immunity, infection was enforced in the waiting area. Weaned piglets with secondary infections received antibiotic treatment.

**Table 1:** Clinical presentation, morbidity and mortality in sows and piglets following the introduction of PEDV in the holding. Abbreviations: pp.: post partum, pn: post natum, ap: ante partum, wk.: week

Group	Sows (morbidity; diarrhea)	Piglets (morbidity; diarrhea)	Suckling piglet losses (mortality, total)	Piglet rearing losses (mortality, total)
**1 (wk 15/2019)**	**approx. 60%**	**approx. 70%**	**10%**	**approx. 5%**
	disease about 10 days pp	disease about 14 days pn	increased proportion of puny little animals or rather underweight weaners	
**2 (wk 17/2019)**	**approx. 80–90%**	**approx. 100%**	**29.2%**	**approx. 10%**
	disease about 0–1 days pp. (bradytocia)	disease approx. 3 days pn (tend to have more weak live suckling piglets than usual)	increased proportion of puny little animals respectively underweight weaners	
**3 (wk 20/2019)**	**approx. 70%**	**approx. 80%**	**20.2%**	**approx. 8%**
	diarrhea in the waiting stable, disease approx. 1–2 weeks ap	disease approx. 3–5 days pn	increased proportion of puny little animals respectively underweight weaners	

In total, three farrowing groups (sows and suckling pigs) showed clinical signs of PED and increased loss rates in suckling pigs (10 to 30%). Morbidity reached 60 to 90% in sows and 70 to 100% in piglets (see Table 1). Some of the weaned piglets also showed diarrhea, wasting, and growth retardation. The overall losses in piglet rearing rose to 5 to 10% (details see Table 1).

In the fourth farrowing group (approx. eight weeks after the first clinical signs) no clinical signs indicative for PED were recorded and up to now no further PED suspicions arose. In autumn, five suckling piglets were randomly selected and subjected to necropsy and PED screening. All samples were negative for PEDV. In the connected fattening unit, no PED signs were recorded at any time. Serological checks in the sow rearing unit (separate building) gave negative results.

During the disease event, intensive cleaning and disinfection was carried out in the farrowing unit, on driveways, in the waiting areas, and all related stables. Disinfectants were chosen in accordance with the list recommended by the Germany Veterinary Society (DVG) for enveloped viruses. Purchase of gilts was stopped and replaced by self-remounting.

Follow-up investigations showed that neighboring farms were also affected by PED shortly before the onset in the described farm.

### Laboratory findings

Upon initial confirmation of PED by a private laboratory, fecal samples from five sows and feces and intestines from two affected piglets were sent to the Friedrich-Loeffler-Institut (FLI) for further analyses and next-generation sequencing. Ribonucleic acids were extracted from fecal samples or supernatants from homogenized intestines using Trizol Reagent (LifeTechnologies, Darmstadt, Germany) in combination with the RNeasy Mini Kit (Qiagen, Hilden, Germany) and DNase digestion on the spin column. All RNAs were confirmed to be PEDV positive by RT-qPCR [24, 25].

Subsequently, all samples were subjected to whole genome sequencing and metagenomic analyses using the Illumina MiSeq platform as previously described [26]. In brief, nucleic acids were processed into shotgun DNA libraries and then deep-sequenced. The resulting raw data was taxonomically classified using the Software pipeline RIEMS [27]. The obtained sequence reads were assembled to determine the genomes in full length. All sequences are available from the INSDC databases under study accession PRJEB38314.

**Fig. 1:**
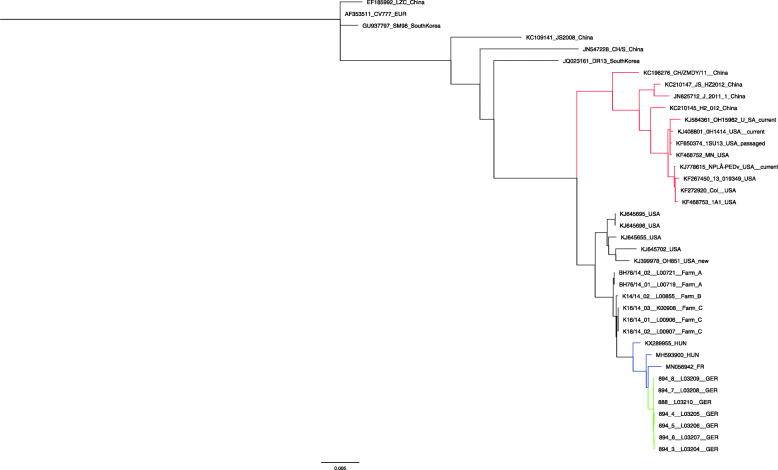
Phylogenetic tree of current PEDV strains. Phylogenetic tree of 2019 PEDV strains from Germany, Hungary, and France as well as 2014 PEDV strains from Germany, USA, and China. The complete genome sequences were aligned using MAFFT and a phylogenetic analysis was performed using PhyML, with a GTR substitution model and tree reconstruction supported by 1000 bootstrapping replicas [28, 29]. Green branches show the 2019 PEDV isolates from Germany, blue branches highlight the isolates from Hungary and France and red branches are the highly virulent NON-INDEL strains from the USA and China

With the help of the Geneious Prime software suite (v. 2019.2.3; Biomatters Ltd., Auckland, New Zealand), phylogenetic analyses were performed (for details see legend Fig. 1). The genomes originating from the reported German case form a new and distinct cluster within the S-INDEL strains (see Fig. 1). Close relatives are three virus strains reported in 2019, two from Hungary [16] (accessions MH593900 and KX289955) and one from France (accession MN056942).

Identity among the new German PEDV strains was almost 100% (> 99.9%) whereas identities of > 98.8% were found with regard to the Hungarian and French sequences. Comparing the German strains from 2014 with the German strains detected in 2019, identities are higher than 99.5%.

Comparisons between German prototype strains from 2014 (the first reported strain, BH76/14-01_L00719_Farm A) and 2019 (894_3_L03204_GER) show high similarities in the nucleotide sequence (see Supplementary Figure 1). In total, 135 nucleotides exchanges are observed over the entire coding region. Of these, 64 nucleotide exchanges are located in the spike protein encoding S gene, especially in its 5′ end, and additional mutations scattered over this coding region. All sequences derived from the case farm showed 100% identity in the S gene. Compared to French and Hungarian strains reported in 2019, identities of > 99.5% were found, while German strains from 2014 were app. 98.5% identical (see Supplementary Table 1). Highly virulent NON-INDEL strains from China and the USA were more distantly related showing identities of app. 95%.

The RNA-shotgun sequencing approach allowed metagenomic analyses using RIEMS. In this analysis, several reads were classified taxonomically as *Picobirnaviridae* sequences. Multiple reads of the RNA-dependent RNA-polymerase gene as well as the gene segment encoding the capsid were found in the fecal but not the intestine samples.

## Discussion and conclusions

Porcine epidemic diarrhea can have a tremendous impact on the pig industry as was seen in the US following the introduction of PEDV in 2013 [2, 29]. Critical losses occurred especially in piglet rearing companies and the losses impacted the whole pork industry [15, 29]. Following the devastating outbreaks on the American continent, re-emergence of PEDV was also reported from Europe after intensified surveillance [23]. However, here, strains of lower virulence were circulating and the reporting and follow-up of cases abated quickly despite ongoing cases. One reason for the subsiding of official follow-up is that PED is neither notifiable nor reportable but still has impact on trade and reputation. Against this background, most farmers had no interest to make their cases public. Thus, official and published information on the German PED situation in general and viral evolution in particular is missing roughly from 2016 onwards.

When PEDV was introduced in a piglet-producing farm in northwestern Germany in 2019, clinical disease and losses were rather disturbing and the farmer and responsible veterinarian initiated a closer follow-up. One hypothesis for the observed impact was a change in virulence and thus, next-generation sequencing was employed to test this hypothesis. Our data show that the causative virus strains are still S-INDEL variants with close relationship to those found in 2014 and the following years. However, viral evolution has taken place and the drift gave rise to a new cluster that comprises recent strains from Germany, Hungary, and France. Given the accordant drift, one can speculate that PEDV is still circulating in Europe. There is no indication that these variants have a higher virulence per se. The previously observed variation seems still present.

The affected farm described in this report was finally able to control the outbreak by forced infection in the waiting unit of sows, biosecurity and strict cleaning and disinfection. Yet, the history of PED in neighboring fattening farms also shows that the virus was able to enter the farm and room for improvement was given in veterinary hygiene and biosafety. The exact route of introduction remained unclear.

Supplementary studies into the metagenomic data set showed picobirnaviral sequences in the fecal material. Picobirnaviruses are non-enveloped double-stranded RNA viruses. They are bisegmented with segment one consisting of 2.3 to 2.6 kilobases and segment two of 1.5 to 1.9 kilobases [30]. Picobirnaviruses are often associated with cases of gastroenteritis or infections in the respiratory tract [31]. The role in diarrhea diseases in piglets is unclear, since picobirnaviruses were found in piglets with and without diarrhea [32]. The transmission is fecal-oral [33] and these viruses have so far been detected mainly in feces in various species [30, 33]. Impact and relevance of these findings remains to be clarified by future studies.

In conclusion, PED re-emerged in northwestern Germany in 2019 leading to high morbidity and substantial impact in a piglet-producing farm. The causative virus strains are still S-INDEL variants but a genetic drift occurred since 2014. This drift is accordant with the evolution in other European countries. The relevance of picobirnavirus detections in fecal samples from PEDV-positive animals remains unclear.

## Supplementary information

**Additional file 1: Supplementary Figure 1.** Whole genome comparison of prototype German PEDV strains from 2014 and 2019. The figure compares the first reported PEDV strain from Germany in 2014 and a representative strain taken from the current outbreak in 2019. Over the whole genome, 135 nucleotide differences are observed that are mainly located in the S-gene (64 out of 135). It can be seen in the magnification of the S-gene and its 5’ end, that changes, i.e. small insertions and nucleotide differences, are mainly located there. **Supplementary Table 1.** S gene sequence identities of recent PEDV genomes. The S gene sequences were aligned using MAFFT from the Geneious Prime software suite (v. 2019.2.3; Biomatters Ltd, Auckland, New Zealand) and sequence identities were exported from Geneious. Green color indicates the strains from the case farm in Germany in 2019, blue sequences mark the PEDV strains from Hungary and France reported in 2019, and representative NON-INDEL strains from the USA and China are highlighted in red.

## Data Availability

Sequence information was deposited at the European Nucleotide Archive (ENA) under study ID PRJEB38314 (Accessions LR812928; LR812932; LR812927; LR812929, LR812930, LR812926, and LR812931). Additional metadata are available from the authors upon reasonable request.

## Abbreviations

PEDV: Porcine epidemic diarrhea virus
PED: Porcine epidemic diarrhea
RT-qPCR: Reverse transcriptase quantitative polymerase chain reaction
kbp: Kilobase pairs
RIEMS: Reliable Information Extraction of Metagenomic Sequence datasets
MAFFT: Multiple alignment using fast Fourier transform
PhyML: Phylogeny software based on the maximum likelihood principle
GTR: General Time Reversible
