# Supplementary material for: Re-emergence of porcine epidemic diarrhea virus in a piglet-producing farm in northwestern Germany in 2019
Source: BMC Vet Res. 2020 Sep 10;16:329. doi: 10.1186/s12917-020-02548-4 (PMC7481547; doi:10.1186/s12917-020-02548-4)
Supplement: Supplementary file 1 — Additional file 1: Supplementary Figure 1. Whole genome comparison of prototype German PEDV strains from 2014 and 2019. The figure compares the first reported PEDV strain from Germany in 2014 and a representative strain taken from the current outbreak in 2019. Over the whole genome, 135 nucleotide differences are observed that are mainly located in the S-gene (64 out of 135). It can be seen in the magnification of the S-gene and its 5’ end, that changes, i.e. small insertions and nucleotide differences, are mainly located there. Supplementary Table 1. S gene sequence identities of recent PEDV genomes. The S gene sequences were aligned using MAFFT from the Geneious Prime software suite (v. 2019.2.3; Biomatters Ltd, Auckland, New Zealand) and sequence identities were exported from Geneious. Green color indicates the strains from the case farm in Germany in 2019, blue sequences mark the PEDV strains from Hungary and France reported in 2019, and representative NON-INDEL strains from the USA and China are highlighted in red. [file 12917_2020_2548_MOESM1_ESM.pptx]

## Slide 1
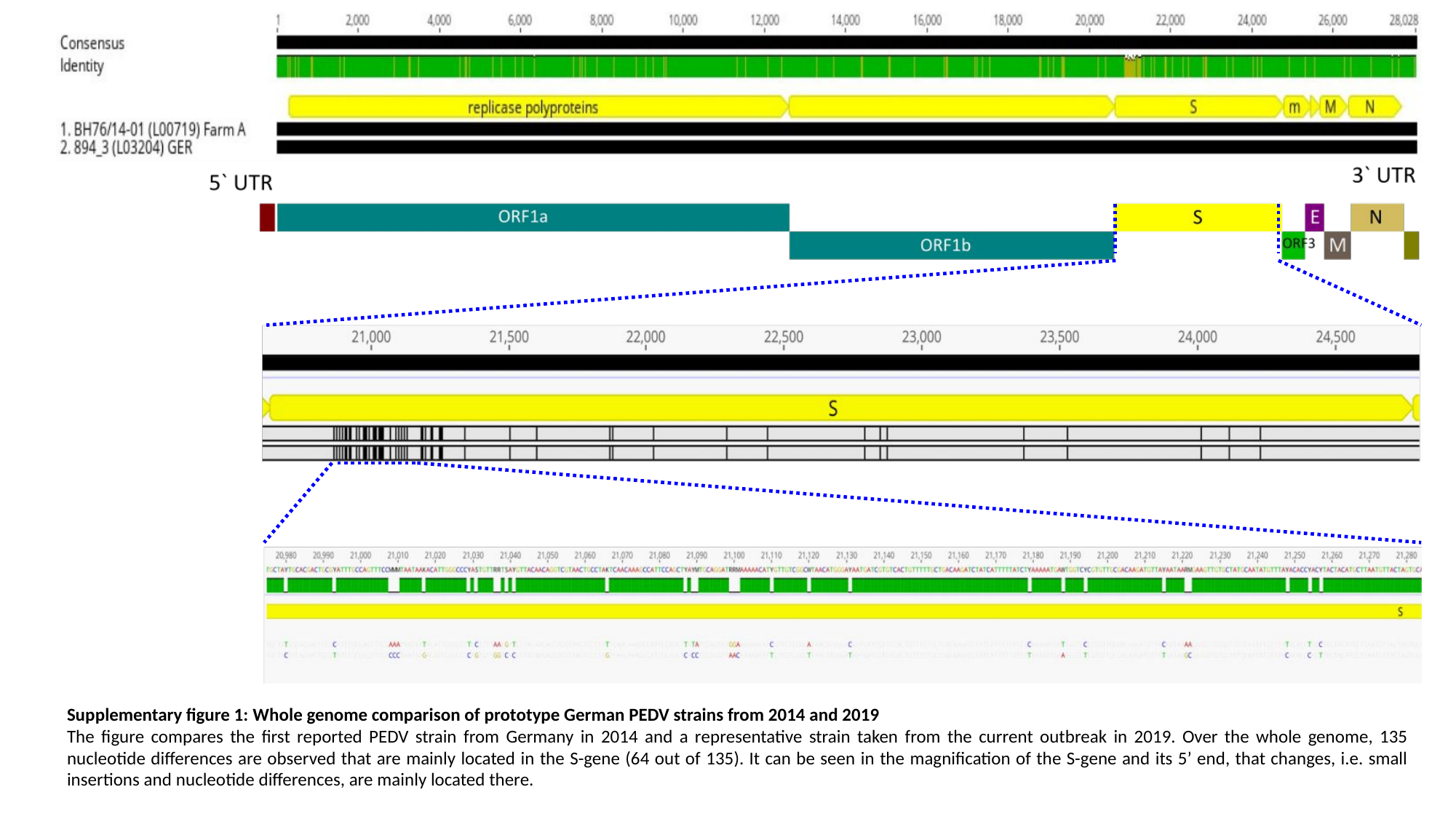

Supplementary figure 1: Whole genome comparison of prototype German PEDV strains from 2014 and 2019
The figure compares the first reported PEDV strain from Germany in 2014 and a representative strain taken from the current outbreak in 2019. Over the whole genome, 135 nucleotide differences are observed that are mainly located in the S-gene (64 out of 135). It can be seen in the magnification of the S-gene and its 5’ end, that changes, i.e. small insertions and nucleotide differences, are mainly located there.

## Slide 2
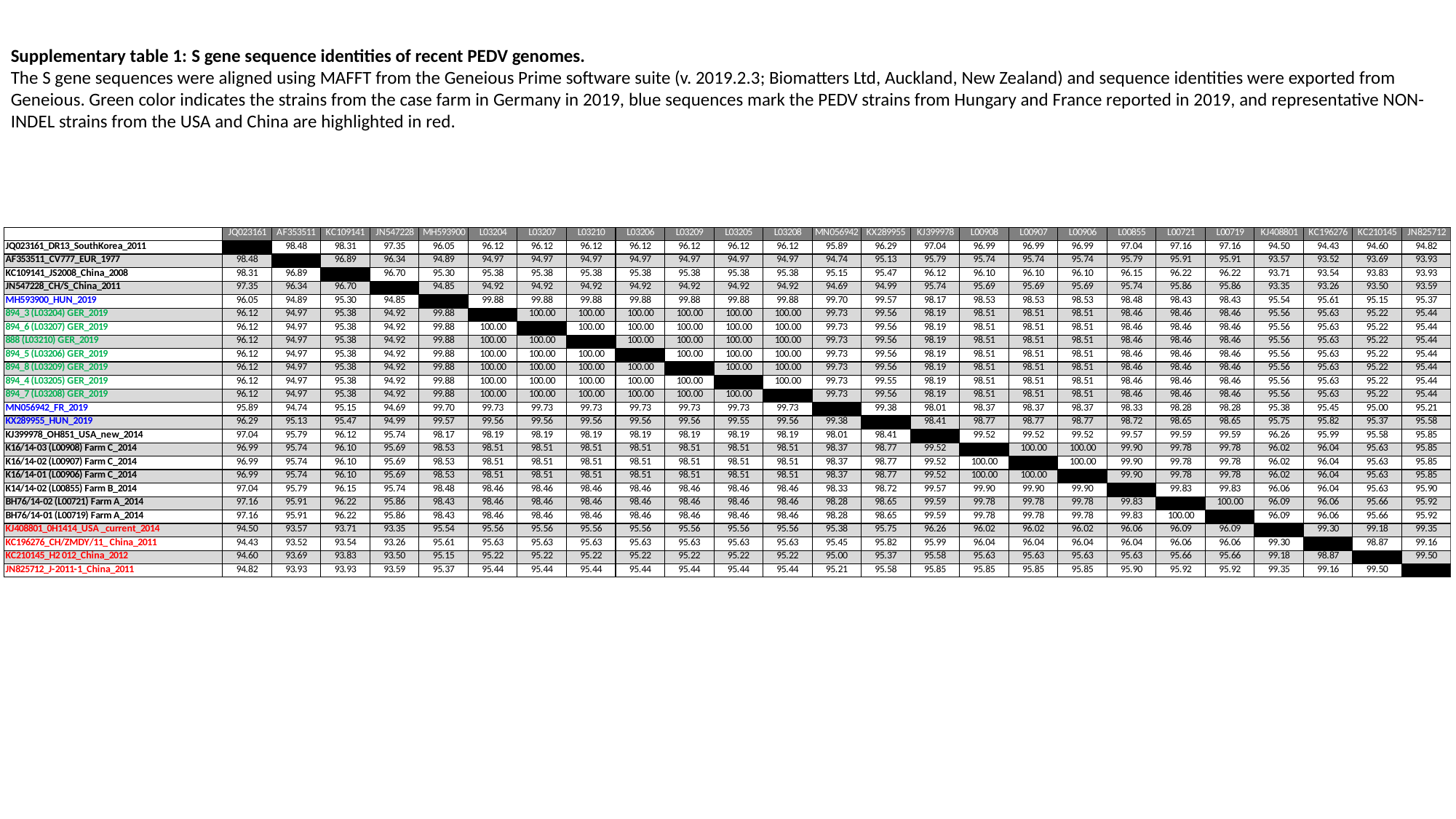

Supplementary table 1: S gene sequence identities of recent PEDV genomes.
The S gene sequences were aligned using MAFFT from the Geneious Prime software suite (v. 2019.2.3; Biomatters Ltd, Auckland, New Zealand) and sequence identities were exported from Geneious. Green color indicates the strains from the case farm in Germany in 2019, blue sequences mark the PEDV strains from Hungary and France reported in 2019, and representative NON-INDEL strains from the USA and China are highlighted in red.
